# A four year longitudinal sero-epidemiological study of bovine herpesvirus type-1 (BHV-1) in adult cattle in 107 unvaccinated herds in south west England

**DOI:** 10.1186/1746-6148-5-5

**Published:** 2009-01-30

**Authors:** Kerry A Woodbine, Graham F Medley, Stephen J Moore, Ana M Ramirez-Villaescusa, Sam Mason, Laura E Green

**Affiliations:** 1 Department of Biological Science, University of Warwick, Coventry, CV4 7AL, UK

## Abstract

**Background:**

Bovine herpesvirus type-1 (BHV-1) is an important pathogen of cattle that presents with a variety of clinical signs, including the upper respiratory tract infection infectious bovine rhinotracheitis (IBR). A seroepidemiological study of BHV-1 antibodies was conducted in England from 2002 – 2004: 29,782 blood samples were taken from 15,736 cattle from 114 herds which were visited on up to three occasions. Antibody concentration was measured using a commercial ELISA. Farm management information was collected using an interview questionnaire, and herd size and cattle movements were obtained from the cattle tuberculosis testing database and the British Cattle Movement Service. Hierarchical statistical models were used to investigate associations between cattle and herd variables and the continuous outcome percentage positive (PP) values from the ELISA test in unvaccinated herds.

**Results:**

There were 7 vaccinated herds, all with at least one seropositive bovine. In unvaccinated herds 83.2% had at least one BHV-1 seropositive bovine, and the mean cattle and herd BHV-1 seroprevalence were 42.5% and 43.1% respectively. There were positive associations between PP value, age, herd size, presence of dairy cattle. Adult cattle in herds with grower cattle had lower PP values than those in herds without grower cattle. Purchased cattle had significantly lower PP values than homebred cattle, whereas cattle in herds that were totally restocked after the foot-and-mouth epidemic in 2001 had significantly higher PP values than those in continuously stocked herds. Samples taken in spring and summer had significantly lower PP values than those taken in winter, whereas those taken in autumn had significantly higher PP values than those taken in winter. The risks estimated from a logistic regression model with a binary outcome (seropositive yes/no) were similar.

**Conclusion:**

The prevalence of BHV-1 seropositivity in cattle and herds has increased since the 1970s. Although the study population prevalence of BHV-1 was temporally stable during study period, the associations between serological status and cattle age, herd size, herd type, presence of young stock and restocked versus continuously stocked herds indicate that there is heterogeneity between herds and so potential for further spread of BHV-1 within and between herds.

## Background

Bovine herpesvirus type-1 (BHV-1) is a member of the family *Herpesviridae*, subfamily *Alphaherpesvirinae*. It is an important pathogen of cattle worldwide [1]. Infection with BHV-1 causes a variety of clinical diseases including infectious bovine rhinotracheitis (IBR) (BHV-1 subtypes 1 and 2a), infectious pustular vulvovaginitis, infectious pustular balanoposthitis (BHV-1 subtype 2b) and encephalitis (BHV-1 subtype 3) [2]. Conventional serological assays cannot distinguish between antigenic serotypes of BHV-1.

BHV-1 generally infects cattle greater than 6 months of age once maternal immunity has waned [3]. Clinical signs associated with infection include nasal discharge, conjunctivitis, fever, inappetance, milk drop, abortion and, sometimes, death, although sub-clinical infection is possible [4,5]. The virus is shed in secretions from the eyes, nose and reproductive organs. After initial infection and disease, cattle become carriers of the virus which becomes latent in the trigeminal or sacral ganglia. Reactivation of the virus may occur when cattle are stressed [4] and virus can then be transmitted to susceptible cattle. Infection can occur indirectly through contaminated material and wind borne particles [2].

BHV-1 has been in Great Britain (GB) since the 1960s [6]. IBR was first confirmed in GB in 1961 [7,8], but was not considered clinically important in GB until an outbreak of disease in Scotland in the late 1970s caused by a virulent strain of BHV-1 (subtype 1) [9-11]. In England and Wales, the overall prevalence of BHV-1 seropositive cattle was 2.1% in 1963 [6], rising to 15.1% of dairy and 16.8% of suckler cattle over 2 yrs of age in Norfolk in 1991 [12], and by 1992 BHV-1 antibodies were detected in 34% of cattle herds in the UK [13].

The reported risk factors for the presence of BHV-1 antibodies in cattle in the Netherlands included a large herd size, dairy herds with beef/veal cattle, a high density of herds in the municipality [14], purchasing cattle, cattle participating in shows, professional visitors not using farm protective clothing and herds situated close to other BHV-1 positive herds [15]. In Belgium, seropositivity was associated with increasing cattle age and herd size and purchased cattle had a higher probability of being infected than homebred cattle [16,17].

This paper presents the largest longitudinal study of BHV-1 seropositivity in cattle in the UK, and its association with cattle age, birthplace, herd type and size.

## Methods

### Source of data

The data used in this paper came from 114 dairy and/or suckler herds in south west England. Farms were visited between 2002 and 2006 and were located within the Randomised Badger Culling Trial (RBCT) [18]. In addition, the study included some farms that had been depopulated in 2001 as a result of the foot-and-mouth disease (FMD) epidemic [19] and subsequently repopulated. All the farmers agreed to participate and allow their breeding cattle to be sampled.

Samples of blood (up to 10 ml) were collected under Home Office licence from all accessible cattle over 2 years of age at three routine visits, at approximately one year intervals. A subset of herds (n = 15) had a whole herd test, i.e. blood samples were collected from cattle of all ages, prompted by the presence of an animal persistently infected (PI) with bovine viral diarrhoea virus (BVDV). Four herds had a whole herd test instead of a routine third visit; otherwise whole herd tests were additional to routine tests.

All blood samples were centrifuged at the University of Warwick at 3220 g for 15 minutes, and serum was removed and frozen at -20°C.

Farmers completed an interview questionnaire between 17^th ^June 2003 and 18^th ^February 2004 to obtain information on clinical signs of infectious bovine rhinotracheitis (IBR) disease and use of a BHV-1 vaccine. Four out of 114 farmers did not complete the questionnaire.

At each visit, the unique identity of cattle was recorded from their ear tag and linked to the relevant blood sample. When appropriate, freeze brand numbers were also recorded. The ear tag was matched to the individual cattle data on the British Cattle Movement Service (BCMS) to obtain/confirm the following: the date of birth, whether the animal was homebred or purchased, breed and sex. More than 99% of cattle were matched within the BCMS dataset. The main reason that cattle were not matched was because they were born before 2001, when it was not compulsory to record cattle birth dates. Herd size was estimated from the cattle tuberculosis testing data (VetNet database), and validated during farm visits.

All data were entered into a relational database (PostgreSQL, PostgreSQL Global Development Group) using Microsoft Access (Microsoft Corp. US) as a front end. All data were screened for errors. When data mismatches were detected, data were re-checked to determine the source of the mismatch and where possible this was corrected.

### Serological test and interpretation

The presence of BHV-1 specific antibodies (IgG_1_) was detected using the SVANOVIR^® ^IBR-Ab (Svanova Biotech AB, Uppsala Sweden) indirect enzyme-linked immunosorbent assay (ELISA). All testing was completed according to the kit instructions. Internal controls were included on each plate to control for batch to batch variation. All samples were run in duplicate, and all testing was completed at the University of Warwick. Positive samples were re-tested when the duplicates were more than 0.25 optical densities (OD) units apart or when the OD of the positive controls was more than 0.2 OD units apart. The sensitivity and specificity of the serological kit to the serum neutralisation test have been reported as 97.4% and 92.4% respectively [20]. BHV-1 antibody concentration was calculated as the percentage of the positive control serum (PP) using the following calculation:

$$pp_{Sample}=\frac{OD_{Sample}}{OD_{PositiveControl}}$$

The PP values ranged from -1.01 to 2.81. A sample was defined as positive when the OD > cut-off and negative when the OD < cut-off. The cut offs were:

Cut-off = 0.2            when *OD*_*Negative control *_× 2.5 < 0.2

Cut-off = *OD*_*Negative control *_× 2.5      when *OD*_*Negative control *_× 2.5 ≥ 0.2

### Vaccination

It was not possible to differentiate antibodies stimulated through vaccination from those from natural infection and so the 7 vaccinated herds were excluded from the analysis.

### Datasets

The full serological dataset consisted of 29,782 ELISA results. Date of birth and identification of cattle were not available for 1,838 and 344 observations respectively. For the purpose of this study, two datasets (A and B) were used. Dataset A included all serological results for all cattle from herds that were not vaccinated against BHV-1 for all visits (26,918 samples, 14,243 cattle, 107 herds). Dataset B was a subset of Dataset A and included all serological results from cattle >2 years of age from the three routine visits and 4 whole herd visits (24,182 samples, 12,764 cattle, 107 herds).

### Outcome variables

A herd was defined as BHV-1 seropositive when at least one cow in the herd was above the cut off for BHV-1 on at least one occasion.

Cattle were defined as BHV-1 seropositive when above the cut off for BHV-1 at least once.

The herd BHV-1 seroprevalence was calculated at each visit as the number of seropositive cattle in the herd divided by the total number of cattle tested.

Seroconversion was defined in a herd when at least one cow changed from being seronegative to seropositive between two visits.

Two outcomes were modelled to investigate associations between BHV-1 seropositivity and herd and cow variables. These were the continuous outcome PP, and a binary (positive/negative) outcome (seropositive yes/no).

### The explanatory variables

The variables listed in Table 1 were tested as fixed effects. The variables triplet code (i.e. the form of badger control in the RBCT), herd restocking status after the 2001 FMD epidemic and farm geographical location were forced into the models because they formed part of the study design.

**Table 1:** The explanatory variables in the repeated measures multi-level model (Tables 3 and 4).

Explanatory variable	Variable defined
Triplet Code	Treatment in the Random Badger Culling Trial (RBCT)
Restocked	Herd was depopulated due to the foot-and-mouth (FMD) epidemic in 2001
Geographical area (farm location)	Geographical area, which the farm was situated in, categorised as Area A-(Gloucestershire, Herefordshire/Worcestershire), Area B-(northeast Devon, south Somerset), Area C-(northwest Devon, northeast Cornwall)
Cattle age (years)	In yearly intervals from 2 years old up to 10 years and over
Herd size	Taken from the VetNet database the average number of cattle present in the herd during the study period
Birthplace of replacement cattle	Cattle were homebred (tested in natal herd) or purchased (tested in herd different from natal herd)
Dairy cattle	Yes or no dairy cattle were present in the herd
Grower cattle	Yes or no grower cattle were present in the herd
Season	Time when visit took place. Winter (December, January and February), Spring (March, April and May), Summer (June, July and August) and Autumn (September, October and November)

### Statistical analysis

Associations between the outcomes and herd and animal factors were initially screened using Mann-Whitney and chi-squared tests. Variables with a significance value of <0.20 in the univariate analysis were tested in two multi-level models: one with a continuous outcome of PP value and one with a binary outcome of seropositive yes/no. There were three hierarchical levels in the models: repeated measure at each visit (level 1), clustered by cow (level 2), and herd (level 3) to control for the dependency between repeated measures within cattle and cattle within herds. The multivariable models were built using both manual forward selection and backwards elimination.

The models took the form:

(1) *Y*_*ijk *_= *βX*_0 _+ *βX*_*k *_+ *βX*_*jk *_+ *βX*_*ijk *_+ *ν*_*k *_+ *u*_*jk *_+ *e*_*ijk*_

where *Y*_*ijk *_is the PP value or seropositivity value at visit *i *from cow *j *in herd *k*. *βX*_0 _is the intercept, *βX *is a series of vectors of fixed effect varying at the herd (*k*), cattle (*jk*) and visit (*ijk*), *ν*_*k *_+ *u*_*jk *_are the variances at the herd and cattle levels, and *e*_*ijk *_is the residual variance.

The model fit was assessed by plotting the raw, standardised and deletion residuals, and the leverage and influence for each random effect and all fixed effects were also assessed [21]. The detection of outliers in the models was completed using a top-down approach [22]: outliers at level 3 (herds) were examined first, and then the lower level clusters (cattle and visits). A model which included only the seropositive herds in Dataset B was also built using the procedure above.

Correlations between the explanatory variables were investigated using chi-squared tests. Variables that were significantly (P < 0.01) associated with each other were noted to help with interpretation. All analyses were done using MLwiN version 2.02 (Centre for Multilevel Modelling, Bristol, UK) [23].

## Results

### Descriptive statistics

#### Study visits

All 107 unvaccinated herds were visited at least once, 96 were visited twice and 95 were visited on three occasions, generating 9136, 8099 and 7850 serum samples, respectively. A further 1833 blood samples were collected from additional visits or because of a whole herd test and/or cattle were under 2 years of age.

#### Vaccination and clinical signs

The median herd size of vaccinated herds was 274; in contrast, the median herd size of unvaccinated herds was 185. The median herd size of unvaccinated seronegative and seropositive herds was 58 and 193, respectively. Nine percent (10/114) of farmers reported that they had seen clinical signs of IBR in their herd in the past, and two of these farmers also reported that they now vaccinated their cattle. All vaccinated herds had at least one seropositive animal compared with 83.2% of unvaccinated herds with at least one seropositive animal. The mean within-herd seroprevalence in vaccinated herds was 74.9% compared with 43.2% in unvaccinated herds.

#### Herd and cattle BHV-1 seroprevalence

There were 89 herds (83.2%, 95% CI, 77.6% – 88.8%) with at least one BHV-1 seropositive bovine on at least one occasion (Table 2). A total of 6,993 cattle (49.0%, 95% CI, 48.2% – 49.8%) always tested negative, and 6,056 always tested positive (42.5%, 95% CI, 41.7% – 43.3%) (Table 2). Nine hundred and eighty five cattle from 53 herds seroconverted between one test and the next, indicating transmission of virus within these herds. The mean and median herd BHV-1 seroprevalence were 43.2% and 40.0% (range, 0.9% – 99.2%) (Table 2). When only the herds where seroconversion had occurred were analysed, the mean and median within herd seroprevalence were 52.9% and 57.1% (range, 2.1% – 99.2%) (Table 2). There was a large statistically significant difference between the median seroprevalence in herds where cattle had seroconverted (57.1%) and those where they had not (10.6%): *U *= 1908; P <0.05, (Mann-Whitney *U*-test).

**Table 2:** Number and percentage of seropositive BHV-1 herds and cattle by visit type for Dataset A (26,918 samples, 14,243 cattle, 107 herds), and herd BHV-1 antibody seroprevalence for positive herds only.

Visit	Positive herds		Positive cattle		Herd seroprevalence for positive herds		
	
	Number	%	Number	%	Median	Mean	Prevalence range
1^st ^routine visit	85	79.4	4,228	46.3	40.0 (51.7)	42.8 (50.9)	0.9 – 95.9 (2.1 – 95.9)
2^nd ^routine visit	74	77.1	3,918	48.4	45.9 (60.2)	43.1 (52.7)	2.7 – 95.9 (2.9 – 95.9)
3^rd ^routine visit/3^rd ^routine and whole herd visit	74	77.9	4,495	50.0	40.4 (59.9)	43.6 (55.1)	2.4 – 99.2 (2.4 – 99.2)
Other non-routine visits	5	38.5	342	49.2	86.6 (73.2)	77.8 (73.4)	43.8 – 100.0 (43.8 – 100.0)
Combined	89	83.0	7,250	51.0	74.0 (74.0)	74.0 (74.0)	73.1 – 75.0 (73.1 – 75.0)

The results for herds where cattle seroconverted are in brackets.

Given the reported sensitivity and specificity of the ELISA, approximately 700 and 2,046 cattle tests during the study are expected to be false negatives and false positives, respectively. There were 124 cattle that tested positive and then negative at subsequent visits. In addition, 85 cattle changed serological status twice: 25 cattle tested positive, negative then positive and 60 cattle tested negative, positive then negative. Note that we assume that antibody positivity remains for life after infection; if antibody titre wanes with time since infection, then a conversion from positive to negative might reflect a true status rather than a test failure.

#### Univariate associations with BHV-1 antibody seroprevalence

**Figure 1:**
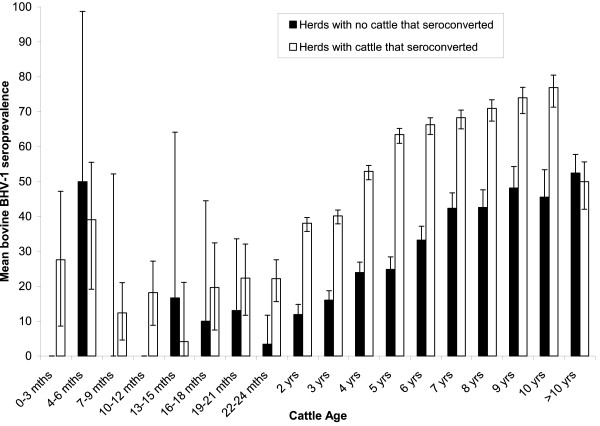
**Unadjusted age-specific profile of the mean BHV-1 antibody seroprevalence and 95% CI by quarter year (up to 2 years old) and year (after 2 years old) for seropositive herds**. Herds are dichotomised by presence of seroconversion in the period of study. All cattle greater than 10 years of age were categorised in one age group. Data came from all visits to BHV-1 unvaccinated herds (Dataset A, see methods section).

**Figure 2:**
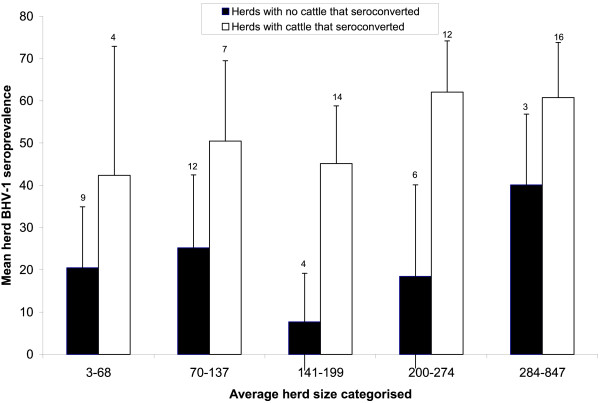
**Mean herd BHV-1 seroprevalence and 95% CI for seropositive herds by herd size (Numbers indicate the number of herds in each category)**. Herds are dichotomised by presence of seroconversion.

The seroprevalence of antibodies against BHV-1 increased with age (Figure 1). The seroprevalence was consistently higher in adult cattle in herds where there was seroconversion but the same trend with age was present in seropositive herds where no cattle seroconverted (Figure 1). The decline in antibody from 6 months of age and seroconversion at approximately 13 months of age is indicative of loss of maternal immunity followed by active seroconversion (Figure 1). The seroprevalence of antibodies was higher in large herds where cattle seroconverted compared with large herds where no cattle seroconverted. There was also a trend that seroprevalence was higher as herd size increased in herds where cattle seroconverted; this was not apparent where there was no seroconversion (Figure 2). There was a statistically significant difference between the medians of the two groups in Figure 2 (*U *= 0; P < 0.05, Mann-Whitney *U*-test).

**Figure 3:**
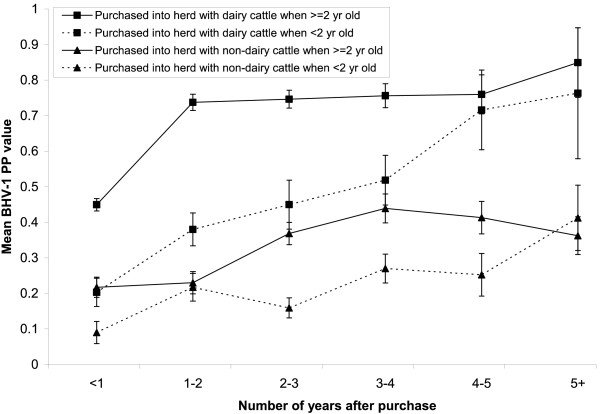
**Mean and 95% CI BHV-1 PP value by the number of years after purchase for age group at purchase and herd purpose**.

Of the 3,452 cattle with no data on their place of birth, 2,101 (60.9%) were seropositive. Of the 10,791 cattle with data, purchased cattle were significantly more likely to be seropositive (54.1%) than homebred cattle (43.6%). The mean PP value was higher for cattle purchased into dairy herds, and increased at a greater rate over time from purchase in cattle ≥ 2 years of age at purchase (Figure 3). There was a highly significant departure from homogeneity between the four categories in Figure 3, *χ*^2 ^= 12137, P < 0.01. There was a significant association between season of visit and PP values. Samples taken in the spring and summer had significantly lower PP values than samples taken in winter; whereas those taken in autumn had significantly higher PP values than those taken in winter.

There was no significant association between restocked/continuously stocked herds and herd seroprevalence in the univariate analysis: 16.7% of restocked herds and 16.9% continuously stocked herds had no seropositive cattle. The herd type with the highest seroprevalence was dairy cattle only (Table 3). There were no herds with dairy cattle and young stock (e.g. dairy with grower herds) that were seronegative.

**Table 3:** Descriptive statistics of the type of herd and number of BHV-1 seropositive unvaccinated herds (Dataset A, see methods section).

				Cattle prevalence in positive herds		
				
Herd type	Mean number of cattle present in herd	No. positive herds	Total No. herds	Mean	Median	Range
Suckler herd	1–100	10	18	16.8	7.2	2.5–74.1
Suckler herd	>100	13	16	38.2	34.0	2.3–80.5
Dairy herd	1–100	5	6	27.4	17.3	7.8–58.7
Dairy herd	>100	35	36	53.1	57.7	0.5–95.4
Suckler with grower herd	1–100	4	7	44.4	49.9	2.7–75.3
Suckler with grower herd	>100	11	11	35.9	34.8	2.6–72.6
Dairy with grower herd	1–100	0	0	-	-	-
Dairy with grower herd	>100	9	9	27.2	18.6	1.9–73.0

Four herds had missing average herd size from the interview questionnaire.

#### Hierarchical model results

There were 19,633 samples (level 1), from 10,039 cattle (level 2) in 102 herds (level 3) with no missing values. In the continuous outcome model there was a significant increase in PP value with age. Cattle from herds with >200 cattle had higher PP values compared with those from herds with ≤ 200 cattle, and there were higher PP values in herds that were formed by restocking after the FMD epidemic of 2001, compared with herds that were continuously stocked. Herds with dairy cattle had higher PP values compared with those without dairy cattle. Herds with grower cattle had lower PP values compared with those without grower cattle (Table 4). In contrast to the univariate results, probably because of the relationship with the restocking variable, purchased cattle had a lower PP value than homebred cattle. The samples collected in spring and summer had significantly lower PP values than those collected in the winter, whereas those taken in the autumn had significantly higher PP values than those taken in winter. There were no significant interactions. The same fixed effects were associated with PP values in seropositive herds only (data not shown). When a variable indicating that cattle on a farm had/had not seroconverted was included (as in Figures 1 and 2) in the final model, all other variables became non-significant.

**Table 4:** Repeated measures multi-level model of 10,039 cattle from 102 unvaccinated herds with PP BHV-1 levels as the continuous outcome and seropositive yes/no as the discrete outcome (Dataset B, see methods section).

					**Continuous**			**Discrete outcome**		
**Variable**	**Category level**	**No. herds**	**No. cattle**	**No. obs**	**Coef**	**SE**^b^	**P value**	**Coef**	**SE**^b^	**P value**

*Intercept*					-0.05	0.08		2.95	0.42	

Triplet code^*a*^	Reactive	35	3839	7286						
	Proactive	31	4900	8660	-0.17	0.09	0.05	-0.43	0.46	0.35
	Survey	36	3665	7776	-0.10	0.09	0.28	-0.19	0.48	0.69
Restocked	No	82	10235	19398						
	Yes	24	2278	4433	0.23	0.07	<0.01	1.10	0.36	<0.01
Farm location	Area A	33	2736	4975						
	Area B	62	8644	16438	-0.01	0.09	0.91	-0.53	0.47	0.26
	Area C	11	1133	2418	-0.01	0.13	0.94	-0.51	0.72	0.48
Age (years)	2	91	1901	1969						
	3	101	3996	4401	0.08	0.01	<0.01	0.16	0.07	0.02
	4	102	3877	4325	0.24	0.01	<0.01	0.72	0.07	<0.01
	5	100	3239	3634	0.35	0.01	<0.01	1.11	0.07	<0.01
	6	101	2652	2870	0.41	0.01	<0.01	1.36	0.08	<0.01
	7	96	2011	2181	0.48	0.01	<0.01	1.51	0.08	<0.01
	8	96	1520	1658	0.53	0.02	<0.01	1.59	0.10	<0.01
	9	94	1001	1118	0.59	0.03	<0.01	1.67	0.16	<0.01
	>10	88	641	1675	0.50	0.08	<0.01	0.52	0.70	0.46
Herd size	3–68	22	644	1246						
	70–137	22	1488	2944	-0.02	0.09	0.88	-0.05	0.50	0.92
	141–199	20	2118	4232	0.05	0.09	0.59	0.88	0.49	0.07
	200–274	19	2921	5969	0.25	0.10	<0.01	1.53	0.52	<0.01
	284–847	19	5233	9331	0.40	0.10	<0.01	2.21	0.53	<0.01
Herd had	No	53	3049	6362						
dairy cattle	Yes	53	9482	17469	0.18	0.06	<0.01	1.02	0.33	<0.01
Herd had	No	79	10092	19019						
grower cattle	Yes	27	2439	4812	-0.12	0.07	<0.01	-0.29	0.35	0.41
Cattle birthplace	Homebred	83	5654	11059						
	Purchased	82	3894	7545	-0.06	0.01	0.07	-0.01	0.06	0.87
Season	Winter	55	4008	6862						
	Spring	52	4715	8166	-0.07	0.01	<0.01	-0.25	0.06	<0.01
	Summer	26	2342	3331	-0.02	0.01	0.05	-0.09	0.08	0.326
	Autumn	31	2829	4095	0.10	0.01	<0.01	0.59	0.07	<0.01

^a^See Table 1 for variable definition

^b^Standard Error

The random effects of the repeated measures multi-level model (Variance and SE) Herd: 0.07 and 0.01; Cattle: 0.14 and 0.003; Visit: 0.08 and 0.001.

The sum of the level 1, level 2, and level 3 variances was 0.28. The greatest unexplained variance in PP values in the final multi-level model was between cattle, making up 48% of the total variance. However, the variances were significant at all three levels (Table 4).

**Figure 4:**
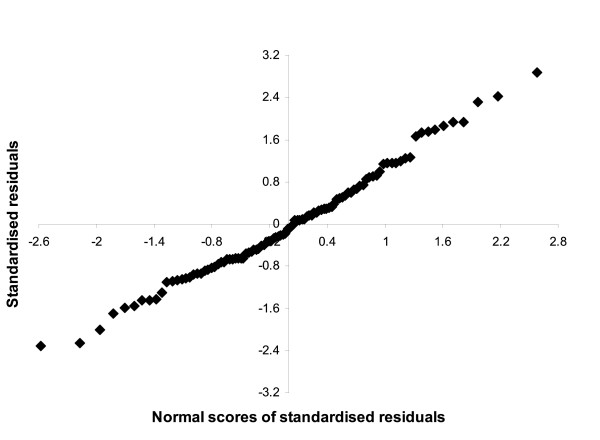
**Normal probability plot of the standardised residuals for the herd level in Model 1 where all unvaccinated herd were included Table 3**.

The normal probability plots of the standardised residuals for all three levels were linear (e.g. Figure 4), indicating that the normality assumption was met. The deleted residuals did not differ greatly from the respective standardised residual values, indicating that there were no large outliers in the data at any level. When the herds, cows or samples with the largest influence were removed, the fit of the model worsened but the results of the model remained unchanged.

The estimated risks were similar when a multi-level binary logistic regression model (outcome seropositive yes/no) was fitted (Table 4). The variables that did not remain significant were whether cattle were homebred or purchased and whether herds had grower cattle.

## Discussion

This is the largest seroepidemiological study of BHV-1 carried out in cattle herds in the UK, and the first for more than 15 years. The seroprevalence of BHV-1 has increased dramatically since the 1960s in both cattle and herds. In the 1960s the seroprevalence in cattle was 2.1% [6], whilst in the current study, 42.5% of cattle always tested positive. Similarly, the herd prevalence was 20% in 1974 [24], and 83.2% in the current study. The differences in herd seroprevalence between the current and previous studies could be explained in part if the herds in this study were biased towards a particular location, age or cattle purpose since these were factors that influenced herd seroprevalence (Table 4). However, the consistency of seropositivity across the farms in this study over the 3 year time period suggests that infection has reached a temporal equilibrium, or possibly that seropositivity is increasing slowly.

A particularly interesting result is the higher seroprevalence in herds that were restocked after FMD. Carrique-Mas et al. (2008) [25] reported that bovine tuberculosis could be introduced into a farm at restocking when cattle were bought from herds with high incidence of infection. It is highly likely that purchase will have brought BHV-1 into restocked herds. The reason for the apparent increase in seroprevalence of BHV-1 is unknown but it might be that movement and mixing in new herds is stressful for cattle, resulting in recrudescence of virus in infected cattle that then infect susceptible cattle in the newly formed herd. In non-restocked herds, purchased cattle had a lower seroprevalence, which is contradictory to this explanation. It should also be remembered that the vaccination status of individual cattle is unknown so that the difference in seroprevalence might be explained by differences in purchasing vaccinated cattle, especially in dairy herds. There may also be complex interactions between cattle age, purchasing and restocking, since restocking of herds is very different to "routine" purchase. Additionally, the existence of multiple antigenic types (which cannot be distinguished serologically) means that interpretation of seroepidemiology is not straightforward. Whatever the cause, these results suggest that these purchased cattle were associated with a high herd seroprevalence and highlight the point that farmers should consider the antibody BHV-1 status of cattle before introduction, to prevent concomitant introduction or re-introduction of BHV-1 of the same or a different antigenic type, that might influence disease presentation.

The seroprevalence of BHV-1 increased with herd size in the univariate analysis and in the hierarchical model; this is consistent with other studies [16,26-28]. The association with herd size was greater in herds where seroconversion occurred during the study (Figure 2), suggesting that active transmission is related positively to herd size, perhaps because larger herds have more potential transmission contacts both within the herd and with other herds (e.g. from veterinarians, other farmers and purchased cattle). However, the association between BHV-1 PP values and herd size might be confounded with other factors associated with herd size, e.g. recrudescence of infection through stress [29], or exposure to more viral types.

The age serological profile is consistent with life-long seropositivity. Antibodies to BHV-1 in calves of ≤ 6 months of age probably correspond to colostrum derived maternal immunity. This wanes by 1 year of age, and starts to rise by 2 years of age because of active infection. This suggests that the optimum age for vaccination of cattle is 10 – 14 months of age, although with some active seroconversion observed in adult cattle and a seroprevalence only exceeding 50% in older animals, vaccination is likely to have a positive impact when administered at any age. All the vaccinated herds had seropositive cattle, while approximately 80% of the unvaccinated herds had seropositive cattle. On average, less than 50% of the cattle in the unvaccinated herds tested positive at each visit, whilst >75% of the cattle from vaccinated herds were positive. These data suggest that either some cattle in vaccinated herds were not vaccinated, that vaccination does not always induce antibody or that antibody titre induced by vaccination wanes over time. It is possible that antibody titre generated by natural infection also wanes over time, but there is no previous work on this hypothesis.

In the current study, when the herd was a dairy herd rather than a suckler herd, the rate of seroconversion in adults was greater. The average age of cattle did not differ between dairy and suckler herds, and so this is a true effect, possibly due to winter housing or other stressors related to dairy cow management.

Housing and winter stressors are also an explanation for the small but significant effect of season on PP values. However, 31% and 47% of visits were 11–13 months apart for routine visits 1 and 2 and routine visits 2 and 3, respectively, so that there was a tendency for the same herds to be sampled in the same season and so herd effect is interpreted as an apparent seasonal effect. Other explanations are that seasonal culling and replacement of cattle might influence herd prevalence of BHV-1 antibodies.

All the variables above became non-significant when the binary variable seroconversion (indicating whether seroconversion was observed in the herd) was introduced into the final model. This indicates that the model risk factors are those associated with virus circulation between cattle on the farm.

The use of different modelling approaches has been discussed elsewhere [28,30] but not modelling different distributions of the same outcome. We compared these two outcomes because we wanted to investigate change in antibody level that was not necessarily occurring around the cut off. The associated risks were similar to those in Table 4 whether the data were modelled using a continuous or binary outcome, but the presence of grower cattle and purchased or homebred were not statistically significant in the binomial model, although the trends were in the same direction of risk. This suggests that these variables alter the antibody titre either always above or below the cut off and that this change was detectable in the continuous outcome model but not in the discrete model.

In the discrete model, we assumed that the serological measurement of seropositive/seronegative had perfect sensitivity and specificity. It is possible that bias could be introduced into the analysis if the sensitivity or specificity of the test varied with other variables, for example, with cattle age, or time since infection, however this has not been reported for BHV-1 antibody tests. The continuous outcome (PP) calculated from the ELISA will have had some random error (Figure 4), but avoided the need to categorise cattle as positive or negative to BHV-1 antibody, and avoided the possibility of misclassification due imperfect sensitivity and specificity, although would have retained a bias if serological measurement varied with other variables. However, the consistency between the discrete and continuous outcome models suggests that sensitivity and specificity do not alter with any of the explanatory variables in the model.

The results of this study do not necessarily represent all cattle herds in England, because of the locality of the farms sampled and because the analysis was based primarily on the serological values for BHV-1 from adult cattle. Using only unvaccinated herds in the study may have reduced the extrapolation of the results, because these herds may have had different management practices from vaccinated herds, but there were only 7 herds that reportedly used vaccination and so analysis of these herds' managements was not possible. In addition, there might have been an overestimation of the infection derived seroprevalence because purchased cattle might have been sourced from a vaccinated herd and so had antibodies derived from vaccination: we do not know the vaccination status of source herds. The serological test used in the current study did not distinguish between vaccine derived and pathogen derived antibody so this was unavoidable. It is interesting that only 7 herds were vaccinated and that farmers reported little clinical disease from BHV-1, suggesting that this infection is not an obvious production constraint or clinical disorder in most herds.

Finally, in the current analysis we could not distinguish between BHV-1 sub-types, the epidemiology of individual types might be different and this might account for some of the unexplained heterogeneity. BHV-1 subtype 3 is now classified as a new species (bovine herpesvirus-5 or BHV-5) [31]. It is probable that we were detecting antibodies to BHV-5 as well as BHV-1 because these viruses are antigenically related.

## Conclusion

In conclusion, the level of BHV-1 infection has increased dramatically since previous estimates from the UK, with approximately 83% of the unvaccinated herds in the south west of England seropositive. This study adds to previous work with the finding that infection might have reached a temporal but not a spatial equilibrium and that between herd variability in PP might arise through variability in cattle age, herd size, herd purpose and the formation of new herds.

## Authors' contributions

KAW helped collect samples, performed the data analysis and drafted the manuscript. GFM and LEG are the funded co-applicants who designed the study, supervised data collection and analysis and finalised the manuscript. SJM coordinated and completed ELISA testing. ARV assisted in design of the questionnaire and coordinated data collection and supervised collection of blood samples. SM setup and maintained the study database and downloads. All authors read and approved the final manuscript.
